# Methylation of tumour suppressor genes APAF-1 and DAPK-1 and *in vitro* effects of demethylating agents in bladder and kidney cancer

**DOI:** 10.1038/sj.bjc.6603482

**Published:** 2006-11-28

**Authors:** F Christoph, C Kempkensteffen, S Weikert, J Köllermann, H Krause, K Miller, M Schostak, M Schrader

**Affiliations:** 1 Department of Urology, Charité – Campus Benjamin Franklin, Universitätsmedizin Berlin, Hindenburgdamm 30, Berlin 12200 Germany; 2 Institute of Pathology, University Hospital Hamburg-Eppendorf, University of Hamburg, Berlin, Germany

**Keywords:** hypermethylation, p53 target gene, p53 mutation, zebularine, 5-aza-2′-deoxycytidine

## Abstract

To examine the significance of the methylation level of the p53 target and tumour suppressor genes apoptotic protease activating factor-1 (APAF-1) and death-associated protein kinase-1 (DAPK-1) in 80 microdissected tumour samples from transitional cell carcinoma (TCC) of the bladder and 80 tumour samples from clear-cell renal cell carcinoma (RCC) as well as from non-tumourous bladder and kidney tissue. Growth-inhibitory effects of the demethylating agents 5-Aza-2′-deoxycytidine (5-Aza-CdR) and zebularine were investigated in TCC and RCC cell lines. The methylation frequency of APAF-1 (DAPK-1) was 100% (77%) in TCC and 100% (33%) in RCC. The methylation levels of APAF-1 could differentiate between the individual tumour stages in TCC as well as in RCC. The APAF-1 methylation levels in RCC were significantly higher in tumours larger than 4 cm and in high-grade tumours. The methylation frequencies in normal tissue for APAF-1 (DAPK-1) were 11% (8%) in bladder tissue and 9% (5%) in kidney tissue. The growth-inhibitory effect of the demethylating agents in TCC (RT4, T24) and RCC (A498, ClearCa-5) cell lines resulted in a 17–132% prolongation of the doubling time (DT). In RCC cell lines, zebularine was superior to 5-Aza-CdR in achieving a DT prolongation. Quantitative real time RT-PCR detected a re-expression of mRNA transcripts of APAF-1 or DAPK-1. In conclusion, demethylating agents effectively retard growth of TCC and RCC cell lines. Methylation level analysis of specific genes has the potential for further tumour characterisation in TCC and RCC.

Bladder and kidney cancers are among the most frequent urogenital malignancies with an annual incidence of 91 000 for bladder and 37 000 for kidney cancer in the European Union (Boyle and Ferlay, 2005). The majority of bladder tumours (70%) appear to be superficial, but a number of them will eventually recur and some will progress to higher stages. Once the tumour becomes muscle-invasive, radical surgical measures like cystectomy are recommended. Systemic platinum-based chemotherapy can be applied in the adjuvant or neoadjuvant setting of locally advanced or node-positive bladder cancer or in cases of metastatic disease (Garcia and Dreicer, 2005). Renal cell carcinoma (RCC) is not sensitive to chemo- or even radiotherapy and immunotherapeutic approaches using cytokines are limited in effectivity and in the survival advantage they provide in metastatic disease (Amato, 2000). Recently, multikinase inhibitors, such as sunitinib or sorafenib, have demonstrated efficacy and disease-stabilising activity as single agents in second-line therapy for patients with cytokine-refractory metastatic clear-cell RCC (Motzer *et al*, 2006; Ratain *et al*, 2006).

One mechanism in the multistep model of tumorigenesis is the promoter methylation of specific tumour suppressor genes. If methylation occurs within the promoter region of a suppressor gene, epigenetic silencing of this gene may lead to functional inactivation, a mechanism reported for various tumour entities (Jones and Baylin, 2002).

If the methylation of a tumour suppressor gene is relevant for gene silencing, the reversal of methylation by demethylating agents could lead to reactivation of the gene. Unfortunately, the applicability of the commonly used 5-Aza-2′-deoxycytidine (5-Aza-CdR) is hampered by its high toxicity and instability in physiological solutions (Momparler, 1985; Bender *et al*, 1998). However, the recently identified zebularine is less toxic and more stable and can be administered orally (Kim *et al*, 1986; Cheng *et al*, 2003).

Deregulation of apoptosis leads to irregular cell survival and has been implicated in the development of cancer. Functional loss of the proapoptotic p53 gene has been recognised as a common event in a wide range of cancer types (Levine *et al*, 1991). However, only a few mediators of apoptosis, especially the p53 target genes, have been shown to relate gene inactivation with tumour progression (Feinstein *et al*, 1995; Jia *et al*, 2001; Soengas *et al*, 2001; Tada *et al*, 2002).

The aim of this study was to further investigate the occurrence and relevance of promoter methylation of the p53 target genes death-associated protein kinase-1 (*DAPK-1*) and apoptotic protease activating factor-1 (*APAF-1*) in relation to different stages of tumour progression in transitional cell carcinoma (TCC) and RCC. In addition, *in vitro* experiments were conducted with the demethylating agents 5-Aza-CdR and zebularine to investigate their global growth inhibitory effects in different bladder (RT4, T24) and kidney cancer (A498, ClearCa-5) cell lines with p53 wild-type (RT4, A498) and p53 mutated (T24, ClearCa-5) status.

The two genes were assessed for their methylation status in microdissected tissue by quantitative real-time methylation-specific polymerase chain reaction (PCR) (MSP), applying the normalised index of methylation (NIM). Quantitative real-time RT-PCR was used to investigate the effects of the demethylating agents on subsequent mRNA re-expression following treatment with 5-Aza-CdR or zebularine.

## MATERIALS AND METHODS

This study included 80 patients (35 women and 45 men) with superficial and muscle-invasive bladder cancer disease and 80 (24 women and 56 men) who had primary clear-cell RCC and underwent radical nephrectomy. All patients were treated at the Department of Urology between January 1997 and December 2000. The bladder cancer patients were submitted to transurethral resection of the bladder for superficial disease and to radical cystectomy after initial transurethral resection for muscle-invasive disease. Normal transitional cell tissue was obtained from 20 patients who underwent open surgery for benign prostatic hyperplasia. Normal kidney specimens were obtained by nephrectomy for a nonmalignant tumour disease like hydronephrosis in 10 cases and by taking nonmalignant kidney tissue distant from the tumour in 10 other cases. The tissue samples were shock-frozen immediately after surgical resection of the tumour or normal tissue and stored in liquid nitrogen at −80°C. Before further processing, samples were serially sectioned, stained with hematoxylin-eosin and examined by a uropathologist (JK) to ensure that slices used for subsequent DNA extraction mainly contained malignant tissue of the respective tumour. The areas with the highest neoplastic cell content (>80%) were selected and microdissected with a sterile needle under microscopic control. The TNM classification of the International Union against Cancer was used for histopathological staging and grading (1997). All patients signed a consent form approved by the Committee on Human Rights in Research at our institution.

### DNA isolation and preparation

After dissection, DNA was isolated from the tissue using the BioRobot® EZ1 workstation according to the manufacturer's instructions (Qiagen, Hilden, Germany). The DNA concentration was determined by spectrophotometry, and its integrity was checked by 1.5% gel electrophoresis.

### Bisulfite modification and MSP

A total of 2 *μ*g of genomic DNA from cell line and tissue samples was subjected to bisulfite modification using the MethylEasy™ DNA Bisulfite Modification Kit (Human Genetic Signatures Pty Ltd, Macquarie Park, Australia) according to the manufacturer's instructions. Methylation analysis was performed by fluorescence-based real-time PCR using Taqman hybridisation probes with the Light Cycler Instrument (Roche, Germany). Each primer set contained at least two CpG sites and was located within the promoter region of the gene. Polymerase chain reaction consisted of initial denaturation for 10 min at 95°C, followed by 50 cycles with individual annealing temperatures and times (*APAF-1* 60°C, 20 s; *DAPK-1* 58°C, *MyoD* 59°C, 15 s) and final elongation for 40 s at 72°C. Polymerase chain reaction was also performed for non-CpG-containing regions of *MyoD*, which served as a control gene (sense: 5′-CCA ACT CCA ATT CCC CTC TCT AT, antisense 5′-TGA TTA ATT TAG ATT GGG TTT AGA GAA GGA, Taqman 6FAM-5′-TCC CTT CCT ATT CCT AAA TCC AAC CTA AAT ACC TCC XT--PH; acc. no. AF027148) (Jeronimo *et al*, 2001). The primer sequences for *APAF-1* were (sense: 5′-TTT CGG GTA AAA GGG ATA GAA TTA GA, antisense 5′-AAA AAT CTT CCC GAC CTA TAA CGC, Taqman 6FAM-5′-ATA CCG CTA CGA CAC CTC AAA TCT TCG C--TMR; acc. no. AB070829). For *DAPK-1* they were (sense: 5′-TCG TCG TCG TTT CGG TTA GTT, antisense 5′-TCC CTC CGA AAC GCT ATC G, Taqman 6FAM-5′-CGA CCA TAA ACG CCA ACG CCG--TMR; acc. no. NM_004938). For relative quantification, standard curves were generated separately for *APAF-1*, *DAPK-1* and *MYOD1* from serial dilutions of bisulfite-modified CpGenome™ Universal Methylated DNA (Chemicon International, Temecula CA, USA), and standards were included in each PCR run. The NIM was calculated for each sample as described elsewhere (Gonzalgo *et al*, 2004).

### RNA isolation, preparation and real-time RT-PCR

Total RNA was extracted from cell lines and human testicular samples using RNeasy® Mini Kit (Qiagen GmbH, Hilden, Germany), according to the manufacturer's instructions. RNA concentration was determined using NanoDrop ND-1000 and RNA integrity was approved using the Agilent Bioanalyzer 2100 (Agilent Technologies, Waldbronn, Germany). Quantitative real-time RT-PCR for *APAF-1* and *DAPK-1* mRNA (genbank acc. no. AF013263 and NM_004938) was performed on LightCycler® system. In a one-step RT-PCR reaction, 250 ng of total RNA were subjected to cDNA synthesis and subsequently amplified during 35 PCR cycles (reverse transcription: 10 min at 55°C, reverse transcriptase inactivation: 30 s at 95°C, cycles: 0.5 s at 95°C, 15 s at 60°C, 20 s at 72°C) using LightCycler® RNA Amplification Kit Hybridisation Probe (Roche Diagnostics GmbH, Penzberg, Germany). The specific primers used for mRNA amplification were as follows: *APAF-1* forward: 5′- GCT CTC CAA ATT GAA AGG TGA AC-3′; reverse: S5′- ACT GAA ACC CAA TGC ACT CC-3′; FL-probe: 5′-AAA AGG GAA TGA TCT CTA ACA GCT TCT GCA-FL; LC-probe: 5′-LC Red640: CTA ATA CAG ACT TCC CAC AGC CTG CCA-PH and *DAPK-1* forward: 5′-AAA ACC ACC CTT GTA GAA TCT CTC AA-3′; reverse: S5′-GTT CTC GCA GCC TGG GTA C-3′; FL-probe: 5′-CTT TTT CAG AAG GCG TCG GCC C-FL; LC-probe: 5′-LC Red640: GAC TGT CTT CCA CCA ACT CCA GCA GGT T-PH. In parallel, RT-PCR detecting the reference gene porphobilinogen deaminase (PBGD) was performed for each sample using the LightCyler® h-PBGD Housekeeping Gene Set according to the manufacturer's recommendations (Roche Diagnostics, Mannheim, Germany). For relative quantification, standards were established from serial dilutions of *APAF-1*, *DAPK-1* and *PBGD* cDNA templates, generated separately and included in each run. Relative gene expression (RGE) was calculated for each sample, as the ratio of *APAF-1* or *DAPK-1* copy number (target gene) to PBGD mRNA copy number multiplied by 100, thus normalising *APAF-1* or *DAPK-1* mRNA expression for sample to sample differences in RNA input, quality and PCR efficiency (Karge *et al*, 1998).

### Cell culture and drug treatments

The human bladder carcinoma cell lines RT4 and T24 were grown in RPMI 1640 medium supplemented with 10% foetal bovine serum, sodium pyruvate and L-glutamine (Rigby and Frankis, 1970). The RCC line A498 (American Type Culture Collection) was cultured in RPMI 1640 medium, supplemented as described above (Giard *et al*, 1973). The RCC cell line ClearCa-5 was derived from a typical representative of the clear-cell type of RCC (Wethkamp *et al*, 2006). Initial drug treatment was started 24 h after seeding. RT4, T24, A498 and ClearCa-5 cells were plated (3 × 10^5^ cells 100-mm^−1^ dish) for continuous drug treatment with zebularine to a final concentration of 10^−4^ M. Treatment was started when cells were in logarithmic growth. The medium was changed every 3 days along with fresh zebularine treatment. RT4, T24, A498 and ClearCa-5 cells were plated for sequential drug treatment with 5-Aza-CdR (3 × 10^5^ cells 100-mm^−1^ dish). Cells were in logarithmic growth when 5-Aza-CdR was added after 24 h to a final concentration of 2 × 10^−6^ M. After 24 h, the medium was changed again, and 5-Aza-CdR was again added for 24 h to a final concentration of 2 × 10^−6^ M. Then the medium was changed and cells were grown in fresh untreated medium. The concentrations of 5-Aza-CdR and zebularine were chosen according to previous reports (Cheng *et al*, 2004a).

The control group comprised cells grown under the same conditions but treated with phosphate-buffered saline (PBS). DNA and RNA were harvested after 7 days for methylation and RT-PCR analyses, respectively. All cells were maintained at 37°C in a humified atmosphere with 5% CO_2_ in air and cultured in RPMI 1640 (Biochrom AG, Berlin, Germany). 5-Aza-CdR (Sigma-Aldrich, A3656) was stored as a 2 mM and zebularine (Sigma-Aldrich, Z4775) as a 10 mM stock solution dissolved in PBS.

### Determination of population doublings and cell growth

Cells were counted with a Z1 Coulter particle counter (Beckman Coulter Corporation, Hialeah, Fl, USA) on the seventh day after the initial treatment. Untreated cells were analysed under similar conditions for control purposes. Each experiment was repeated four times. Doubling times (DT) were calculated 72, 120 and 168 h after treatment according to the following formula: DT=time*X*/[log(*nX*/*n*0)/log2] with *nX* and *n*0 being the cell count at times *X* and 0 (Kraemer *et al*, 2003).

### Statistical analysis

Two-tailed statistical analysis was performed using SPSS computer software (Version 12, SPSS Inc, Chicago, IL, USA). The Mann–Whitney *U*-test was used to statistically evaluate the methylation level of the individual genes in relation to the tumour stage and grade. The paired student's *t*-test was used for evaluation of mRNA expression levels in the *in vitro* studies. For the comparison of cell DTs, statistical analysis was carried out using the Wilcoxon pair-difference test. All *P*-values <0.05 were considered statistically significant.

## RESULTS

This study included 80 patients with TCC of the bladder: 20 pTa tumours, 30 pT1 tumours and 30⩾pT2 tumours. Transitional cell carcinoma was low- or intermediate-grade (G1/2) in 24 patients and high-grade (G3) in 56. Twelve patients with superficial pT1 G3 tumours had received BCG intravesical instillation therapy, and 16 patients with muscle-invasive bladder carcinoma had undergone cisplatin-based chemotherapy. In a control group, the methylation status of normal urothelium was measured in 20 patients without malignant disease. All 80 patients in the group with RCC had clear-cell tumours. Tumours were grouped as pT1 in 37 patients, pT2 in 21 and pT3 in 22. Tumours were G1/2 in 30 patients and G3 in 50. There were 61 (76%) node-negative and 19 (24%) node-positive tumours.

In the patient group with TCC, *APAF-1* was found to be methylated in all samples investigated, corresponding to a methylation frequency of 100%. The methylation frequency of *DAPK-1* was 80% (16 out of 20) in pTa, 80% (24 out of 30) in pT1 and 70% (14 out of 20) in muscle invasive ⩾pT2 tumour disease. The median NIM levels for *APAF-1* (*DAPK-1*) were 22% (31%) in the pTa group, 60% (23%) in the pT1 group, and 78% (16%) in the ⩾pT2 group. When the tumours were grouped according to their differentiation grade, the median NIM was 20% (53.4%) for *APAF-1* in the G1/2 (G3) group and 34.1% (3.6%) for *DAPK-1* in the pTa tumours. In the G1/2 (G3) group of pT1 tumours, the median NIM was 53.5% (71%) for *APAF-1* and 18.3% (24%) for *DAPK-1*. For the muscle-invasive tumour group (⩾pT2, only G3 tumours), the median NIM levels were 97.5% for *APAF-1* and 15.8% for *DAPK-1*. Apoptotic activating factor protein-1 methylation levels were able to differentiate G3 tumours from pTa G1/2 tumours (*P*=0.002) but not from pT1 G1/2 tumours (*P*=0.4). The *APAF-1* NIM levels could also differentiate between the individual tumour stages (*P*<0.001). This was not the case for *DAPK-1* methylation levels. Table 1 gives further details on the median NIM levels.

In the patient group with RCC, the methylation frequency was 100% for *APAF-1* irrespective of the tumour stage. Death-associated protein kinase-1 was found to be methylated in 35% (13 out of 37) of the pT1, 33% (seven out of 21) of the pT2, and 32% (seven out of 22) of the ⩾pT3 tumours. Comparing the median NIM levels of *APAF-1* (*DAPK-1*) revealed a range extending from 41.5% (17.4%) for pT1 to 58% (10.8%) for pT2 and 66% (19.3%) for ⩾pT3 tumours. When the NIM levels were compared according to the specific tumour grade of G1/2 or G3, the median NIM levels for *APAF-1* in G1/2 (G3) tumours were 34% (51%) in pT1, 55% (58%) in pT2, and 72% (66%) in ⩾pT3 tumours. The median NIM levels for *DAPK-1* in G1/2 (G3) tumours were 3% (0%) in pT1, 1% (7%) in pT2, and 0% (13%) in ⩾pT3 tumours. A trend towards a statistically significant correlation was detected by comparing the median NIM levels of *APAF-1* and the different tumour stages (*P*=0.06 and 0.05). The NIM levels could not distinguish between the different tumour grades. Table 2 gives more details.

To exclude a potential bias through age dependency and the methylation level, the TCC and RCC groups were divided according to the median age of the different cohorts, which was 72±11.8 years in the TCC group and 65±12.2 years in the RCC group. Transitional cell carcinoma patients had median NIM levels of 58% in the younger group and 65% in the older group for *APAF-1*, the corresponding levels being 16 and 10%, respectively for *DAPK-1* (*P*=0.4 and=0.8). Renal cell carcinoma patients had median NIM levels of 51% in the younger group and 53% in the older group for *APAF-1*, the corresponding levels being 2 and 2%, respectively for *DAPK-1*. A significant correlation was not detected (*P*=0.4 and 0.6).

The median NIM levels in the patient group with RCC were compared between larger (⩾4 cm) or node positive tumours and smaller (<4 cm) or node negative tumours. The median methylation levels for *APAF-1* were 51% in node-negative tumours and 58% in node-positive tumours, the corresponding NIM levels for *DAPK-1* being 2 and 9%, respectively. A statistically significant correlation was not detected (*P*=0.09 and 0.26). The median NIM levels for *APAF-1* were 42% in smaller tumours and 60% in larger ones; the corresponding levels for *DAPK-1* were 2 and 3%, respectively. A significant correlation was detected for the methylation levels of *APAF-1* (*P*=0.008) but not for those of *DAPK-1* (*P*=0.8).

Comparing RCC specimens according to their differentiation grade revealed a correlation between higher (G1/2) and lower (G3) differentiation; *APAF-1* methylation levels compared as median NIM levels were 41% for high-grade and 55% for low-grade tumours (*P*=0.05).

Finally, the methylation frequency and methylation levels were analysed in normal tissue and methylation frequencies were 11% for *APAF-1* and 8% for *DAPK-1* in bladder tissue and corresponding frequencies of 9 and 5%, respectively in kidney tissue. The median NIM levels were 0% for *APAF-1* and 0% for *DAPK-1* in bladder tissue and 0 and 0%, respectively in kidney tissue (further details see Tables 1 and 2). Also, a dependency between the *APAF-1* and *DAPK-1* methylation levels and the age of the patients was not observed. Median age was 73±5.3 years in the bladder tissue group and 67±6.7 years in the kidney tissue group.

Continuous zebularine or sequential 5-Aza-CdR treatment was applied in two bladder cancer cell lines (RT4, T24) and two RCC cell lines (A498, ClearCa-5). In the bladder cancer cell lines, 5-Aza-CdR treatment retarded the growth of all cell lines investigated. This was demonstrated by a significant increase in DT starting 5 days after the first exposure. Continuous zebularine treatment also retarded the growth of both cell lines; a significant influence was reflected in the prolonged DT found 5 and 7 days after the initial treatment in T24 and RT4, respectively. In both RCC cell lines, 5-Aza-CdR as well as zebularine caused significant growth inhibition, which became apparent on the fifth day after the initial treatment (Table 3).

Treatment with 5-Aza-CdR (zebularine) decreased the median NIM level of *APAF-1* from 144 to 43% (73%) in RT4 and from 138 to 38% (37%) in T24. A comparable effect was observed in the RCC cell lines, where the NIM level of *APAF-1* decreased from 178 to 45% (39%) after treatment with 5-Aza-CdR (zebularine) in A498 and from 149 to 37% (31%) in the ClearCa-5 cell line. Methylation of the *DAPK-1* gene promoter was not observed in any of the four untreated or treated cell lines investigated. The occurrence of NIM levels higher than 100% is attributed to aneuploidy at the gene locus of interest (Friedrich *et al*, 2004).

Quantitative real time RT-PCR was performed to determine whether the observed demethylation effect correlated with an induction of mRNA expression. Apoptotic activating factor protein-1 mRNA expression was detectable in all untreated, methylated control cell lines. After treatment with 5-Aza-CdR the mRNA transcripts of APAF-1 were significantly upregulated in all of the four cell lines. The increase of the median RGE units observed was between 1.5 (ClearCa-5) and 5.4 (RT4)-fold. Exposition to 5-Aza-CdR led to an increase of mRNA expression (in RGEs) of *DAPK-1* that was between 1.7-fold (ClearCa-5) and 11.6-fold (RT4) as compard to the RGEs in the corresponding untreated cell line. Interestingly, the A498 RCC cell line showed a significant decrease of mRNA expression from a median RGE of 60–29 arbitrary units.

After zebularine treatment, a significant upregulation of *APAF-1* mRNA expression was observed in the p53 mutated cell lines ClearCa-5 and T24 only, but not in the p53 wild-type cell lines A498 and RT4. Death-associated protein kinase-1 mRNA expression was – as shown for *APAF-1* mRNA expression – increased in the p53-mutated cell lines ClearCa-5 and T24 but remained relatively unchanged in A498 and RT4. For more details see Table 4.

## DISCUSSION

Recent publications have demonstrated frequent hypermethylation of various genes in urogenital cancers. In addition, the advent of real-time PCR techniques for the quantification of methylation levels has disclosed stage-dependent differences in the methylation levels of the individual genes, as shown for *RASSF1A* and *DAPK-1* in TCC of the upper urogenital tract (Catto *et al*, 2005). Moreover, a few publications, pointing to findings for the DAPK-1 gene, have postulated that the methylation frequency or level correlates with the biological behaviour of the tumour disease (Tada *et al*, 2002; Catto *et al*, 2005). In RCC, various methylation panels have been presented with the highest methylation frequency of 45% for *RASSF1A*, and the *γ-catenin* gene has also been found to correlate with a poorer prognosis (Dulaimi *et al*, 2004; Breault *et al*, 2005).

The role of APAF-1 methylation and its inactivation have been described in malignant melanoma and human leukaemia cell lines. Apoptotic protease activating factor-1 inactivation by promoter methylation was suggested as a factor responsible for the inability of cells to undergo apoptosis (Jia *et al*, 2001; Soengas *et al*, 2001). DAPK-1 is a serine/threonine kinase widely expressed in normal tissue. However, it seems to be epigenetically silenced by promoter hypermethylation, as shown in a variety of cancers, including gastrointestinal, head and neck, and small cell lung cancers and B-cell lymphomas (Kissil *et al*, 1997; Sanchez-Cespedes *et al*, 2000; Kim *et al*, 2001; Chan *et al*, 2005). The imbalance between pro- and anti-apoptotic factors may lead to accumulation of transforming mutations and resistance or decreased sensitivity to anticancer treatment, as shown for *APAF-1* inactivation in human leukaemic cells or *DAPK-1* inactivation in non-small cell lung cancer cells (Jia *et al*, 2001; Tang *et al*, 2004).

Comparing the results obtained from quantitative methylation analysis of the *APAF-1* gene disclosed a high frequency of *APAF-1* methylation. In fact, it occurred in nearly all the bladder and kidney tumour samples investigated. Nevertheless, methylation was also found in normal tissue, though at a lower frequency. Using the NIM enabled us to differentiate not only between benign methylated tissue and malignant tissue but also between lower and higher tumour stages, as NIM levels increased with local tumour progression in TCC. This was also the case in RCC, where tumour characteristics like size and differentiation, but not the nodal status, differed according to the specific *APAF-1* NIM levels. Thus, *APAF-1* methylation levels can help to differentiate between specific tumour stages in TCC and RCC. Death-associated protein kinase-1 methylation was more frequent in malignant than in benign tissue but did not correlate with a higher tumour stage or differentiation grade. Nevertheless, we found a higher frequency of *DAPK-1* methylation in TCC and RCC in a larger cohort than in previous reports (Tada *et al*, 2002; Wethkamp *et al*, 2006). In addition, quantification revealed very heterogeneous methylation levels. No significantly higher methylation levels were detected in muscle-invasive TCC or node-positive RCC. Therefore, *DAPK-1* methylation may not be a prerequisite for a more aggressive tumour phenotype. In a nonquantitative analysis, Tada *et al*, observed a *DAPK-1* methylation frequency of 29% in TCC and found that the recurrence rate correlated with *DAPK-1* methylation in a small cohort of 55 patients (Tada *et al*, 2002). They postulated downregulation of *DAPK-1* by hypermethylation, as immunohistochemical staining was negative in tumour samples with DAPK-1 methylation. Interestingly, Wethkamp *et al* (2006), could not detect methylation of the *DAPK-1* gene in RCC when nonquantitative analysis was performed in a small set of 10 patients. Moreover, they also found persistent *DAPK-1* mRNA expression in a larger cohort of 72 patients with RCC. This suggests post-translational inactivation, as *in vitro* studies in 11 RCC cell lines demonstrated basal DAPK-1 activity in only one cell line despite the detection of *DAPK-1* mRNA expression (Wethkamp *et al*, 2006).

Cheng *et al* (2004a) previously reported growth-inhibitory effects of zebularine in different human cancer cell lines, including T24, a more advanced TCC cell line of the bladder. Moreover, zebularine has been proved to be effective against the development of murine T-cell lymphoma with complete lack of toxicity (Herranz *et al*, 2006). The objective of our study was not only to confirm the effectivity of demethylating agents in growth retardation but also to find possible differences in the treatment response of TCC or RCC cell lines, especially those belonging to a more or less aggressive phenotype. Our data demonstrate for the first time, that both 5-Aza-CdR and zebularine are effective growth inhibitors in non-advanced as well as advanced TCC and RCC cell lines. In TCC, 5-Aza-CdR achieved equal growth inhibition in RT4 and T24 cell lines, which we regard as secondary to its highly toxic effects. Compared to 5-Aza-CdR, zebularine resulted in the same percentage prolongation of the DT in the T24 cell line (79.8 *vs* 79.6%) but not in the RT4 cell line (66.4 *vs* 17.5%). This may be due to the long DT (80 h) of RT4, treatment effects becoming visible after more than 7 days. Another explanation could be the relative resistance of the p53 wild-type RT4 cells to demethylating zebularine treatment, which would necessitate higher doses for equal effectiveness. Demethylating agents are also effective for growth inhibition in RCC cell lines. According to our data, zebularine was an even more effective growth inhibitor than 5-Aza-CdR in the p53 mutated (105.9 *vs* 131.8%) and the p53 wild-type cell line (21.9 *vs* 54.6%). Growth-inhibitory effects of zebularine may be multifactorial, as its DNA and RNA incorporation has been described (Ben-Kasus *et al*, 2005). Microarray experiments have shown increased activation of cancer-related antigens secondary to zebularine treatment, which could result in augmented presentation of cell surface antigens (Cheng *et al*, 2004a, 2004b). Especially in RCC, the combination regimen of zebularine followed by immunotherapy could be a promising treatment option, because it preferentially targets cancer cells, shows activity *in vitro* as well as in experimental animals, and can be orally administered (Marquez *et al*, 2005). We were able to confirm the observations reported by Fu *et al* (2003) who previously demonstrated upregulation of mRNA expression of *APAF-1* in human leukaemia cell lines after exposure to 5-Aza-CdR. However, the reason for the unchanged mRNA expression of *APAF-1* and *DAPK-1* after zebularine treatment in the p53 wild-type cell lines A498 and RT4 is not clear. Zebularine has been shown to be selective towards cancer cells, but is eventually less effective in early stage or less aggressive subtypes to demethylate and subsequently re-express specific, for example tumour suppressor genes such as *APAF-1* (Fu *et al*, 2003; Cheng *et al*, 2004b). It is also not evident, why the unmethylated DAPK-1 gene was re-expressed in the p53-mutated cell lines after exposure to 5-Aza-CdR and zebularine or why 5-Aza-CdR upregulated its expression in RT4 but downregulated its expression in A498 cell lines. Global genome wide effects of these demethylating agents might influence yet unknown regulators of the *DAPK-1* gene, which could be responsible for the effects observed. Therefore, genome-wide analysis could help to further elucidate what is responsible for this differences in regulation of mRNA expression of *DAPK-1*. Moreover, subsequent western blot analysis will have to determine whether there is a correlation between mRNA and protein expression of the *APAF-1* gene. This was not the case in the human leukaemia cell lines studied by Fu *et al* (2003) which points to post-transcriptional regulation of APAF-1 protein expression.

In conclusion, our data present *APAF-1*, a gene frequently methylated in TCC of the bladder and RCC. The *APAF-1* methylation level varies according to the tumour stage in TCC of the bladder. In RCC, *APAF-1* methylation levels depend on specific characteristics such as tumour size or tumour differentiation. Novel demethylating agents seem to be effective for growth retardation not only in TCC but also in RCC cell lines. Thus, the stability and minimal toxicity of the demethylating agent zebularine renders it a promising candidate for epigenetic therapy in RCC and TCC of the bladder.

## Figures and Tables

**Table 1:** Percentage of methylation (NIM) and tumour stage/grade in TCC

	**APAF-1**			**DAPK-1**		
	**G1/2**	**G3**	***P*-value**	**G1/2**	**G3**	***P*-value**
*pTa*						
Median	20	53.4		34.1	3.6	
Mean	22.7	49.6		37.2	17.6	
STD	14.7	17.8		37.1	23.5	
*n*	14	6		14	6	
*P*-value	0.002*			0.6*		
			0.001**			0.8**
*pT1*						
Median	53.5	71		18.3	24	
Mean	62.8	66.4		27.4	41.3	
STD	29.7	19.4		29.1	47.9	
*n*	10	20		10	20	
*P*-value	0.4*			0.7*		
			0.001***			0.09***
*⩾pT2*						
Median	n.o.	97.5		n.o.	1	
Mean		94.1			15.8	
STD		27.1			35.4	
*n*		30			30	

NIM=normalised index of methylation; n.o.=not observed; STD=standard deviation; TCC=transitional cell carcinoma.

* G1/2 *vs* G3 tumours.

** pTa *vs* pT1 tumours.

*** pT1 *vs* ⩾pT2 tumours.

Normal bladder tissue (*n*=20): APAF-1 median (0%), mean (7%), STD (17.3%)/DAPK-1 median (0%), mean (5%), STD (15.5%).

**Table 2:** Percentage of methylation (NIM) and tumour stage/grade in RCC

	**APAF-1**			**DAPK-1**		
	**G1/2**	**G3**	***P*-value**	**G1/2**	**G3**	***P*-value**
*pT1*						
Median	34	51		3	0	
Mean	43.6	44.7		25.6	2.9	
STD	20.9	22.2		39.9	7.9	
*n*	23	14		23	14	
*P*-value	0.6*			0.3*		
			0.06**			0.8**
*pT2*						
Median	55	58		1	7	
Mean	52	56.3		2.3	12.8	
STD	26.9	23.5		3.3	19.6	
*n*	5	16		5	16	
*P*-value	0.8*			0.2*		
			0.05***			0.3***
						
*⩾pT3*						
Median	72	66		*0*	*13*	
Mean	69.3	63.4		0.2	22.6	
STD	26.1	14.3		0.5	24.3	
*n*	2	20		2	20	
*P*-value	0.7*			0.07*		

NIM=normalised index of methylation; RCC=renal cell carcinoma.

* G1/2 *vs* G3 tumours.

** pT1 *vs* pT2 tumours.

*** pT2 *vs* ⩾pT3 tumours.

Normal kidney tissue (*n*=20): APAF-1 median (0%), mean (1.5%), STD (5%)/DAPK-1 median (0%), mean (3%), STD (10.2%).

**Table 3:** DT in hours and prolongation in percent after treatment with PBS, 5-AZA-CdR (2 × 10^−6^ M) and zebularine (10^−4^ M) at various time points (TCC cell lines RT4, T24; RCC cell lines A498, CCa-5)

	**PBS**			**5-AZA**			**ZEB**		
	**DT after**			**DT after**			**DT after**		
	**72 h**	**120 h**	**168 h**	**72 h**	**120 h**	**168 h**	**72 h**	**120 h**	**168 h**
RT4	79.1	75.7	79.4	77.6	83.2*	129.1*	75.4	75.1	88.6*
Percentage		−4.3	+0.4		+7.3	+66.4		−0.05	+17.5
									
T24	19.4	19.5	20.2	19.3	25.5*	34.7*	19.1	22.9*	34.3*
Percentage		+0.5	+4.1		+32.1	+79.8		+19.9	+79.6
									
A498	64.7	65.8	62.9	62.5	71.3*	76.2*	61.5	65.8*	95.1*
Percentage		+2.3	−2.1		+14.1	+21.9		+6.9	+54.6
									
CCa-5	52.7	51.6	57.4	50.6	54.8*	104.2*	53.1	55.6*	123.1*
Percentage		−2.1	+8.9		+8.3	+105.9		+4.7	+131.8

5-AZA-CdR=5-AZA-2′-deoxycytidine; DT=doubling time; PBS=phosphate-buffered saline; ZEB=zebularine.

* =*P*-value <0.05 (Wilcoxon pair difference test).

**Table 4:** RGE of APAF-1 and DAPK-1 mRNA in RCC cell lines A498, ClearCa-5, and TCC cell lines RT4, T24 after treatment with PBS, 5-AZA-CdR (2 × 10^−6^ M) and zebularine (10^−4^ M)

	**APAF-1**			**DAPK-1**		
	**RGE**			**RGE**		
**Cell line**	**PBS**	**5-AZA**	**ZEB**	**PBS**	**5-AZA**	**ZEB**
*A498*						
Median	159	410	139	60	29	58
Mean	164	420	139	60	30	58
STD	24.3	76.7	7.5	2.7	4.4	6.2
*P-*value*		0.002	0.09		0.0006	0.5
						
*CCa-5*						
Median	108	163	133	11	19	20
Mean	110	164	135	11	18	20
STD	14.9	10.2	7.3	3.2	10.3	0.7
*P-*value*		0.003	0.01		0.006	0.007
						
*RT4*						
Median	66	360	65	3	35	5
Mean	65	358	67	3	36	5
STD	2.1	14.3	7.6	0.7	3.8	1.2
*P-*value*		0.0001	0.4		0.0002	0.2
						
*T24*						
Median	188	386	240	0.04	0.5	0.2
Mean	188	386	240	0.04	0.5	0.2
STD	16	5.3	11.1	0.008	0.04	0.01
*P-*value*		0.0001	0.004		0.0002	0.0001

APAF=apoptotic activating factor protein; DAPK=death-associated protein kinase; PBS=phosphate-buffered saline; RGE=relative gene expression; STD=standard deviation; ZEB=zebularine.

* =Comparison between PBS-treated and 5-AZA-CdR- or zebularine-treated cells using paired Student's *t*-test.
